# Comparison of a U.S. and Zambian Ob/Gyn Residency Training Programme

**DOI:** 10.3332/ecancer.2022.1468

**Published:** 2022-11-07

**Authors:** Maya M Hicks, Kenneth Chanda, Mulindi Mwanahamuntu, Monica B Jones, Anissa R Mattison, Krista S Pfaendler, Leeya F Pinder, Groesbeck P Parham, Michael L Hicks

**Affiliations:** 1 Department of Obstetrics and Gynecology, Anne Arundel Medical Center, 2000 Medical Pkwy, Belcher Pavilion, Ste 309, Annapolis, MD 21401, USA; 2 Department of Obstetrics and Gynaecology, University Teaching Hospital – Women and Newborn Hospital, 10101 Nationalist Way, Lusaka, Zambia; 3 Trinity Health Academic Obstetrics and Gynecology, 44428 Woodward Ave Ste 104, Pontiac, MI 48341, USA; 4 Department of Obstetrics and Gynecology, West Virginia University School of Medicine, 64 Medical Center Drive, Morgantown, WV 26506, USA; 5 Department of Oncology, University of Washington, 1959 NE Pacific St, Seattle, WA 98195, USA; 6 Department of Obstetrics & Gynecology, University of North Carolina at Chapel Hill, 101 Manning Dr., Chapel Hill, NC 27514, USA; 7 St. Joseph Mercy Oakland, Michigan Cancer Center, 44405 Woodward Ave, Suite 202, Pontiac, MI 48341, USA; a https://orcid.org/0000-0002-1993-3367; b https://orcid.org/0000-0002-8929-7810; c https://orcid.org/0000-0001-5922-5990; d https://orcid.org/0000-0002-1819-155X

**Keywords:** post-graduate medical education, comparisons of postgraduate medical education, inequities in postgraduate training, U.S. and African postgraduate training, postgraduate training in low- and middle-income countries (LMICs), postgraduate training in Africa, misperceptions in international research collaborations

## Abstract

**Introduction:**

The major objective of the study was to compare and contrast a U.S. and Zambian Ob/Gyn residency programme, using uniform metrics, as the basis for an initial exploration of perceived inequities in post-graduate medical education between low- and high-income countries.

**Methods:**

Measurements of the following procedures were used to indicate whether minimum standards had been met by trainees in their respective postgraduate programmes: vaginal deliveries; C-sections; abdominal, vaginal and laparoscopic hysterectomies; other laparoscopic surgeries; cancer cases; abortions; obstetrical ultrasounds; cystoscopies; incontinence and pelvic floor surgeries. Evaluations were also made with respect to the presence or absence of an official ultrasound rotation, subspeciality and off-service rotations, protected didactic time and exclusive time on obstetrics and gynaecologic clinical services. Comparisons were made relative to these various categories and the average procedural numbers at each level of training to determine differences in trends and degree of exposure.

**Results:**

Minimal procedural requirements were met by both the U.S. and Zambian programmes. For open surgical cases, the minimum standards were higher for the Zambian programme, whereas for procedures associated with the use of high-end technology, such as ultrasound and minimally invasive surgery, minimum standards were higher for the U.S. programme.

**Conclusion:**

There were no significant differences in the Zambian and U.S. Ob/Gyn post-graduate training programmes, relative to their respective metrics. A more extensive analysis is required to determine the actual competency levels that are produced by the respective training systems.

## Introduction

International women’s health research partnerships involving investigators from countries with vastly different income statuses are increasing. It is important that individuals from these different settings understand the quality of professional training of their respective colleagues. Otherwise, misperceptions may arise, resulting in inequities in cooperation [1]. In these circumstances, a comparative analysis of respective postgraduate training programmes can yield information that may help to avoid these misunderstandings and the subsequent negative attitudes they may engender. Investigations of this type are severely lacking in the published literature [2].

In order to address this knowledge gap, we designed an observational study comparing and contrasting a U.S. and African (Zambian) Ob/Gyn postgraduate (residency) training programmes. Our major objective was to determine similarities, variances and inequities in training; and to discern if residency graduates have the same documented capabilities, based on uniform metrics.

## Materials and methods

The metrics of the following minimum procedural and educational standards were compared to determine differences and similarities in the U.S. and Zambian Ob/Gyn residency training programmes: duration (number of years) of the training curriculum; residents per level; presence of official ultrasound rotations; time on obstetrics rotations and gynaecology rotations, exclusively; presence of protected didactic time; subspeciality and off-service rotations; numbers of vaginal deliveries; C-sections; abdominal, vaginal and laparoscopic hysterectomies, other laparoscopic procedures; hysteroscopies; surgery for invasive cancer cases; abortions; obstetrical ultrasounds; cystoscopies; incontinence and pelvic floor surgery [3, 4] (Table 1).

The respective programme’s average minimum procedures were analysed at each level of training to determine differences in trends, exposure to surgical procedures and achievement of minimal targeted standards by the completion of training, as crude measurable determinants of proficiency (Tables 2 and 3). This information was acquired from the data banks of the respective programmes, collected by each respective programme to determine if residents are achieving their minimum required procedural standards [3, 4]. It encompasses all current levels of training, years 1–4, at both programmes.

There was no ethics or internal review board approval needed for this study given that it did not involve confidential patient information or subjects that could be harmed.

## Results

Both the U.S. and Zambian residency training programmes are 4 years in duration. The U.S. programme is accredited through the Accreditation Council for Graduate Medical Education, whereas the Zambian programme is accredited through the Higher Education Authority undertaken at the Department of Obstetrics and Gynaecology, University of Zambia, stationed at the Office of Dean for Postgraduate Medicine. These accrediting bodies also set the required minimal procedural standards for their respective programmes. The Zambian programme’s basic minimum standards were higher than those of the U.S training programme for the following procedures: vaginal deliveries (210 versus 200); C-section deliveries (400 versus 145); abdominal hysterectomies (35 versus 15); cancer cases (65 versus 25); number of abortions (55 versus 20). The U.S. programme’s minimum standards were higher than those of the Zambian programme for numbers of vaginal hysterectomies (15 versus 5); obstetrical ultrasounds (50 versus 40) and cystoscopies (10 versus 5). The Zambian programme has not yet established minimum standards for laparoscopic, hysteroscopic, incontinence and pelvic floor surgical procedures.

Structurally, the Zambian programme has not yet established protected didactic/educational time, nor formal rotations in perinatology/maternal foetal medicine, reproductive endocrinology or breast care services. The U.S. programme has established formal rotations in the aforementioned disciplines and provides 1 half-day/week of protected didactic/educational time. The Zambian programme has 36 months exclusively on obstetrics and gynaecology clinical services, with 1 year (12 months) of off-service rotations, whereas the U.S. programme integrates these off-service rotations during the course of the 48-month training period, resulting in 42 months of clinical Ob/Gyn activities, inclusive of subspeciality clinical rotations (Table 1). The Zambian programme tailored the number of procedures to achieve a targeted aggregate at each level of training, allowing the compulsory number of procedures to be reached by the completion of the training programme; whereas the U.S. programme had much more variability to do as many procedures within each level of training, with no tailored customisation on the numbers of procedures at any level of training in order to reach the targeted requisite number of procedures by the completion of the training programme (Tables 2 and 3).

Both programmes have a requirement for all residents to complete a research project in order to enhance their understanding of the principles of scientific investigation during the 4-year training period.

Target minimum procedural standards were met by the Zambian and U.S. programme in all categories, as determined by their respective sanctioning bodies.

## Discussion

In comparing and contrasting the U.S. and Zambia programmes, it is noted that the minimum procedural standards were higher for the Zambian programme in several areas that required open surgery, such as C-sections, abdominal hysterectomies and surgery for cases of invasive cancer. However, the Zambian programme lacked minimum procedural requirements for minimally invasive surgical procedures (hysteroscopy and laparoscopy) (Table 1). Endoscopic/laparoscopic procedures require expensive equipment as well as additional educational training and systems for ancillary personnel to set up, operate and maintain equipment. It also requires deferred resources for maintenance to sustain the equipment in optimal operational condition, which can be cost prohibitive in resource-constrained settings. While there are proven benefits of minimally invasive surgery, it is important to note that lack of performing endoscopic procedures does not diminish proficiency in the speciality.

Twelve (12) months of the 4-year curriculum in the Zambian programme is spent exclusively on off-service rotations. This allows resident trainees to perform the remaining 36 months of clinical obstetrics and gynaecology without interruptions. This approach theoretically allows trainees to become more clinically proficient through sustained repetition; and this may explain why they have a tailored targeted approach on procedures at each level of training compared to the U.S. programme. The Zambian trainees are exposed to subspeciality training in gynaecologic oncology and also had a higher number of cumulative cancer cases compared to the U.S. programme. This may be explained by the fact that Zambia has one of the world’s highest incidence rates of cervical cancer (26/100,000 Zambia versus 6.2/100,00 U.S.); the training site is also the tertiary referral site for all cancers in the country; and there is a gynaecologic oncology fellowship affiliated with this programme [5]. There are no official perinatology/maternal foetal medicine, female pelvic reconstructive surgery/urogynaecology and reproductive endocrinology rotations. Reproductive endocrinology is offered in the private sector but not in the residency teaching system. All high-risk pregnancies are managed by Zambian obstetric consultants.

The U.S. programme has a more comprehensive structure with more exposure to subspeciality rotations, obstetrical ultrasound, incontinence and pelvic floor surgery and laparoscopic/minimally invasive surgery. These advantages are related to access to more technological advancements (robotics, incontinent sling applications, etc.). Although the resource settings between the two programmes are different and the expectations of the above highlighted procedures are applicable to their respective resource setting, it is of utmost importance to state that the surgical approach, open or minimally invasive, does not change the indication and/or requirements for surgical intervention. Future research investigations that evaluate the impact of minimally invasive surgery on resident education and patient outcomes in resource-constrained settings would be of great interest.

Both programmes are 4 years in length and require the completion of a research project prior to graduation. However, unlike the U.S. programme, the Zambian programme offers a Master of Medicine (MMed), in which graduates are required to complete an official dissertation to receive the master’s degree (Table 4). This certification was created in 1981 to counteract attrition in the speciality and to benefit the recipient by facilitating the opportunity to pursue a Ph.D. While such an opportunity is important in a rigidly structured formal academic setting like Zambia, it is not so in the U.S. system, where multiple and diverse opportunities exist for obtaining a high level of economic security. The two other Zambian Ob/Gyn training programmes are non-MMed and do not represent a potential pathway to a Ph.D. However, since their initiation 10 years ago, both have had great impact relative to building clinical capacity in Zambia.

Both the U.S. and Zambian programmes are led by senior programme directors. They are staffed by full-time and adjunct faculty with experience in both clinical management and research, are extensively published in peer-reviewed journals and serve as clinical and research mentors to their respective residents (Table 4). This allows for residents to have adequate exposure to the fundament principles of research in the event they choose to pursue such endeavours.

We would postulate that, based on the metrics put forth by each respective sanctioning body, Zambian and U.S. obstetrician/gynaecologists are equally trained in all the basic areas of obstetrics and gynaecology, with the former more experienced in open cases and the latter in the use of minimally invasive surgical platforms.

There are only three Ob/Gyn residency programmes in Zambia compared to 286 programmes in the U.S [6]. To date, the Zambian MMed Ob/Gyn programme has produced 90 graduates compared to 200 by U.S. Ob/Gyn programme. This contrast highlights the need to build clinical capacity in women’s healthcare in a country like Zambia (Table 4).

The majority of Zambian Ob/Gyn graduates assume positions as clinical specialists under the Ministry of Health and are posted to various hospitals across the country to work independently, within a few months of programme completion. The majority of graduates from the comparable U.S. programme pursue private or employed hospital based practices, with a small minority of graduates entering into subspeciality training.

In this investigation, we have only focused on the speciality of obstetrics and gynaecology in one African nation, where we have accumulated significant clinical and research experience [7–13]. We are keenly aware that there may be differences in other specialities of medicine. Certainly, there will be variations and differences in African training programmes across the vast continent, as there are in U.S./North America. This variation is undoubtedly a weakness of the observational approach used in this study.

## Conclusion

While basic minimal procedural standards are a reasonable method to use to compare programmes, they alone are not sufficient, by themselves, to make a statement related to overall competency of the graduates completing the programme. Another more in-depth analysis would be required to better determine competency, using other metrics and personal evaluations. However, information emanating from our investigation fills a needed gap in the literature. Hopefully it will stimulate other disciplines to make similar comparisons, leading to a better understanding of the basic educational training of respective groups of colleagues, and thereby avoiding misperceptions and strengthening partnerships in research and clinical collaborations.

## Conflicts of interest

None.

## Funding

None.

## Figures and Tables

**Table 1.** Comparison of basic minimum standards for the respective programmes.

Metric	U.S. Ob/Gyn residency	Zambia Ob/Gyn residency
Number of years in residency programme	4	4
Number of residents per level of training	4	8
Number of vaginal deliveries	200	210 (5% higher)
Number of C-sections	145	400 (63.5% higher)
Number of abdominal hysterectomies	15	35 (57% higher)
Number of laparoscopic hysterectomies	15	-
Number of laparoscopic procedures	60	-
Number of hysteroscopies	40	-
Number of cancer cases	25	65 (61.5% higher)
Number of vaginal hysterectomies	15 (66.6% higher)	5
Number of abortions	20	55 (64% higher)
Number of obstetrical ultrasound	50 (20% higher)	40
Number of cystoscopies	10 (50% higher)	5
Number of incontinence and pelvic floor surgery	25	-
Official ultrasound rotation	X	X
Months on gynaecology	21 Inclusive of 17 months of benign gynaecology (general gynaecology), 2 months of gynaecologic oncology, 1 month of urogynaecology and 1 month of reproductive endocrinology infertility rotations	18 Gynaecologic oncology is the only subspeciality service that is covered simultaneously in conjunction with the benign gynaecology (general gynaecology) service, but there is no separate dedicated rotation
Months on obstetrics	21 Inclusive of 1 month of perinatology/maternal foetal medicine rotation	18 No subspeciality rotation on the obstetrical service
Protected didactic educational time	X	O
Gynaecologic oncology rotation	X	X
Perinatology rotation	X	O
Reproductive endocrinology rotation	X	O
Intensive care unit (ICU) rotation	X	X
Neonatal intensive care unit (NICU) rotation	O	X
Emergency room rotation	X	X
Family planning rotation	X	X
Breast service rotation	X	O
Urogynaecology rotation	X	O
Research requirement and formal instruction on scientific research	X	X

X = yes

O = No

**Table 2.** U.S. programme: procedure averages per year (cumulative per year. Achievement of targeted minimum procedural standards is designated by the respective summation numbers in the 4th year column).

Metrics	1st year	2nd year	3rd year	4th year
Number of vaginal deliveries	78	152	260	258
Number of C-sections	15	55	114	151
Number of abdominal hysterectomies	0.4	0.25	6.25	30
Number of laparoscopic hysterectomies	0.4	0	7.25	38
Number of laparoscopic procedures	1.4	16.5	26.75	82
Number of hysteroscopies	10	36.25	64.50	87
Number of cancer cases	0	0	1.5	34
Number of vaginal hysterectomies	0.2	0.25	3.5	17
Number of abortions	9	11.75	21	20
Number of obstetrical ultrasound	35	164	290	103
Number cystoscopies	2	5	11.25	34
Number of incontinence and pelvic floor surgery	0.6	5.5	10.75	43

**Table 3.** Zambian programme: procedure averages (non-cumulative/tailored/targeted per year. Designation of achievement of targeted minimum procedural standards is derived by the summation of each yearly number in the row of the respective procedural metric).

Metrics	1st year	2nd year	3rd year	4th year
Number of vaginal deliveries	120	50	20	20
Number of C-sections	20	180	120	80
Number of abdominal hysterectomies	-	5	10	20
Number of laparoscopic hysterectomies	-	-	-	-
Number of laparoscopic procedures	-	-	-	-
Number of hysteroscopies	-	-	-	-
Number of cancer cases	5	10	40	10
Number of vaginal hysterectomies	-	-	-	5
Number of abortions (manual vacuum aspirations)	20	20	10	5
Number of obstetrical ultrasound	-	-	40	-
Number cystoscopies	-	-	5	-
Number of incontinence and pelvic floor surgery	-	-	-	-

**Table 4.** Other differences between the respective programmes.

Differences	U.S. programme	Zambian programme
MMed	No	Yes
Number of in-country residency programmes	286	3
Full-time faculty	5	4
Adjunct part-time faculty	30	13
Number of graduates since the beginning of the programme	200	90
Contextual clinical echo system	High-income	Low- and middle-income
